# Facilitators and barriers to uptake and adherence to lifelong antiretroviral therapy among HIV infected pregnant women in Uganda: a qualitative study

**DOI:** 10.1186/s12884-017-1276-x

**Published:** 2017-03-21

**Authors:** Esther Buregyeya, Rose Naigino, Aggrey Mukose, Fred Makumbi, Godfrey Esiru, Jim Arinaitwe, Joshua Musinguzi, Rhoda K. Wanyenze

**Affiliations:** 1 Department of Disease Control and Environmental Health, School of Public Health, College of Health Sciences, Makerere University, Kampala, Uganda; 2 Department of Epidemiology and Biostatics, School of Public Health, College of Health Sciences, Makerere University, Kampala, Uganda; 3 AIDS Control Program, Ministry of Health, Kampala, Uganda; 4 Global Fund Focal Coordination Office, Ministry of Health, Kampala, Uganda

## Abstract

**Background:**

In 2012, Uganda started implementing lifelong antiretroviral therapy (ART) for prevention of mother to child transmission (PMTCT) in line with the WHO 2012 guidelines. This study explored experiences of HIV infected pregnant and breastfeeding women regarding barriers and facilitators to uptake and adherence to lifelong ART.

**Methods:**

This was a cross-sectional qualitative study conducted in three districts (Masaka, Mityana and Luwero) in Uganda, between February and May 2014. We conducted in-depth interviews with 57 pregnant and breastfeeding women receiving care in six health facilities, who had been on lifelong ART for at least 6 months. Data analysis was done using a content thematic approach with Atlas-ti software.

**Results:**

Initiation of lifelong ART was done the same day the mother tested HIV positive. Several women felt the counselling was inadequate and had reservations about taking ART for life. The main motivation to initiate and adhere to ART was the desire to have an HIV-free baby. Adherence was a challenge, ranging from not taking the drugs at the right time, to completely missing doses and clinic appointments. Support from their male partners and peer family support groups enhanced good adherence. Fear to disclose HIV status to partners, drug related factors (side effects and the big size of the tablet), and HIV stigma were major barriers to ART initiation and adherence. Transition from antenatal care to HIV chronic care clinics was a challenge due to fear of stigma and discrimination.

**Conclusions:**

In order to maximize the benefits of lifelong ART, adequate preparation of women before ART initiation and on-going support through family support groups and male partner engagement are critical, particularly after birth and cessation of breastfeeding.

## Background

Mother to child transmission of HIV (MTCT) is the main contributor to the paediatric HIV epidemic [1]. Despite high HIV testing rates among pregnant women, global access to antiretroviral therapy (ART) among HIV positive pregnant women remains unacceptably low, at about 30% [2]. Uganda is among the 22 high- HIV burden countries, which account for about 90% of all pregnant women needing antiretroviral drugs (ARVs) for prevention of mother to child transmission of HIV (PMTCT) [3–5].

The 2013 World Health Organization (WHO) ART guidelines for PMTCT recommend two options, including lifelong ART to all pregnant and breastfeeding women living with HIV regardless of CD4 count or clinical stage [6]. Uganda was among the first countries in Africa to adopt lifelong ART, previously referred to as Option B+ [6]. This choice was based on programmatic and operational reasons, particularly in generalized epidemics, with high fertility, small birth intervals, and poor access to CD4 testing [6]. Lifelong ART provides important clinical and programmatic benefits in that, it is easy to implement, is in harmony with guidelines for ART in non-pregnant adults, prevents MTCT in future pregnancies, prevents sexual transmission and has maternal health benefit [3–6].

Countries that have implemented lifelong ART, such as Malawi and Uganda report a tremendous increase in the numbers of pregnant women enrolled on ART [7]. However, there are concerns about uptake, adherence, retention of women and their exposed infants in care, referral and transition from the PMTCT program to HIV chronic care [5]. A few studies highlight a high rate of loss to follow-up and challenges with linkages across various clinics for women and child services [8, 9]. A systematic review of adherence to ART during and after pregnancy, showed that adherence levels were poor during postpartum period [53.0%, 95% confidence interval (CI) 32.8–72.7%] compared to pregnancy (75.7%, 95% CI 71.5–79.7%) [10]. Understanding the barriers and facilitators of uptake, adherence, and long-term retention is critical to maximizing the benefits of lifelong ART. In this paper, we present findings from a qualitative study conducted to explore experiences of HIV infected pregnant and breastfeeding women regarding barriers and facilitators of uptake and adherence to lifelong ART in Uganda.

## Methods

### Study design

This was a cross-sectional qualitative study using in-depth interviews (IDIs) with HIV infected pregnant and lactating women.

### Study site and population

This study was conducted in three predominantly rural districts including Mityana, Luwero and Masaka. These districts were purposively selected because they were among the first districts to implement lifelong ART in Uganda in 2012. The women were drawn from six health facilities: Mityana Hospital in Mityana District, Luwero Health Centre (HC)IV and Katikamu HCIII in Luwero district, Ssunga HCIII, Kyanamukaka HCIV and Masaka Regional Referral Hospital in Masaka district. The selected facilities were the first to implement lifelong ART in the three districts. Antenatal care (ANC) services in these facilities run daily on weekdays. All mothers who attend ANC are offered an HIV test [11].

### Selection of study participants

IDIs were conducted among HIV positive women, both pregnant and post-partum who had been on lifelong ART longer than 6 months in order to provide longer experiences pre- and post-delivery. The interviews were conducted among three groups of participants: good ART adherers, poor ART adherers and delayed ART acceptors. Good adherers were mothers who were consistent in getting their ART refills for at least 6 months, while the poor adherers were those that were not consistent, based on the mothers’ health facility records. The delayed acceptors were mothers who delayed to start ART i.e., not picking their supply for at least 4 months after being initiated. Using purposive sampling strategy, health providers and expert clients (HIV infected peers) identified women who belonged to the three categories (good adherers, poor adherers and delayed acceptors), contacted them either face-to-face or by telephone and asked if they would be willing to participate in the study. An appointment was set, usually on the next scheduled clinic visit, at home or other convenient location. The research assistants administered informed consent and interviews in a private place to ensure confidentiality. We targeted to interview 10 participants at each of the six facilities (60 women overall).

### Data collection

Based on our research question.i.e., experiences of HIV infected pregnant and breastfeeding women regarding barriers and facilitators to uptake and adherence to lifelong ART we developed an interview guide. The research team discussed the issues/topics to be explored, based on what was lacking in the literature. The topics included; the mothers’ journey towards ART initiation, motivation to start lifelong ART, adherence to ART, facilitators and barriers to adherence. From these topics, the team developed an interview guide which spelt out how the interview was to be conducted and how to respond to the things the respondents say. We developed a list of questions or issues in a semi-structured format to be explored during the interview and the sequence to be followed. In addition, follow-on questions or probes were included where needed. Data collection took place between February and May, 2014. Three research assistants (two females and one male) with substantial experience in qualitative research conducted the interviews. The research assistants were trained on the study objectives and how to administer the interview guide. Each IDI was conducted by one research assistant and audio-recorded, after written consent from the participant. The topics that were discussed consisted of: the mothers’ journey towards ART initiation, motivation to start lifelong ART, adherence to ART, facilitators and barriers to adherence. This was intended to yield information on challenges and good practices experienced by the women to inform interventions to improve ART adherence. The interview guide was reviewed by the whole research team and necessary adjustments were made after pretesting it through role plays and field interviews. The interviews were conducted in *Luganda* the commonly spoken local language, and lasted between 1 and 2 h.

### Data management and analysis

The interviews were concurrently transcribed and translated into English, and imported into Atlas-ti software for coding and analysis. Content analysis was conducted. Transcripts were coded independently by two researchers (EB and RN). Data was organized using existing themes in the interview guide and emerging themes, after reading through the transcripts several times. The authors discussed and reconciled the suggested codes. Related codes were merged into categories, which later formed the themes.

## Results

### Characteristics of study participants

Out of the 60 planned IDIs, 57 were conducted. These included 18 pregnant, 37 lactating women, one woman who had a miscarriage, and another who had lost the baby. Among the enrolled women, 29 were poor ART adherers, 21 were good ART adherers, and seven delayed ART acceptors. The majority of the respondents were young mothers aged 15–24 years (age range16-42 years), Table 1. Most of the women were married (41, 72%), had completed primary education (33, 58%), and had carried a previous pregnancy (54, 95%).

**Table 1:** Socio-demographic characteristics of the study participants

Variable	Number (*n* = 57)	Percentage (%)
Age in years		
15–24	29	51.0
25–34	21	37.0
35+	7	12.0
Education level		
No formal education	5	8.7
Primary 1–7	33	57.8
Senior 1–4	17	29.8
Senior 5+	2	3.5
Marital status		
Married	41	72.0
Not married	16	28.0
Number of previous pregnancies		
0	3	5.3
1 to 2	25	44.0
3+	29	51.0
Adherence category		
Good adherers	21	36.8
Poor adherers	29	50.9
Delayed adherers	7	12.3

Interviews from the pregnant and breastfeeding women showed similar views; therefore, the presentation of results is integrated. Where necessary, the similarities or differences across participant categories and study sites are highlighted. The data are presented according to the themes that emerged and illustrative quotes from respondents are used to exemplify the results. The women’s experiences are organized across two major themes including 1) ART initiation and 2) Adherence to ART including barriers and facilitators.

### ART initiation

Three main subthemes emerged under this theme, including women’s experiences during ART initiation, facilitators and barriers of ART initiation.

### Women’s experiences during ART initiation

Majority of the mothers started taking ARVs at ANC the same day they tested positive for HIV. Some mothers had initiated ART before they became pregnant. In one facility, women were given an option to defer the ART if they were not ready or did not want to start treatment. Almost all the mothers reported no formal checks for preparedness to start ART (e.g., disclosure, previous adherence history in relation to other drugs, treatment supporter or pre-treatment counselling). The women felt that the intention of the health workers was to enroll as many women on treatment as possible, with little effort on counseling and health education.

*“… After giving me my results, she came and gave me the medicine. She told me that ‘those who are supposed to educate you more are not around. Let me give you this medicine and you go, come on this day you will find the other health workers, who will explain to you.’ I was not educated on anything”* Poor adherer, Mityana Hospital

*‘As long as they test you and you are found with the HIV virus, you start on the medicine (ART) on that day.’* Poor adherer, Ssunga HCIII

### Facilitators for initiating ART among HIV infected pregnant women

The desire to have a healthy baby (HIV-free baby) was expressed by almost all the mothers, including good and poor adherers, as the main motivator for starting ART. Other motivating factors included: the need to remain healthy and live longer so as to take care of their children. Some participants reported having been motivated to start treatment by people they know who have lived long with HIV because they are on treatment while others cited people who had died because they did not take their treatment, which they referred to as “*carelessness*”. Some mothers feared being recognized as HIV infected because of the emaciation due to HIV as a motivator for ART initiation. One mother reported having been motivated by a health worker to start treatment. She previously refused to take ARVs while pregnant because she did not have any symptoms such as fever or headache. On the subsequent pregnancy, she was still tested and told that she was HIV positive and was convinced to take ART by health workers, after testing positive the second time.

‘…*, when they told me the first time, I became afraid…but I had neither pain, nor high temperature. I was not feeling anything. I waited to experience a fever, headache, or pain, but it did not happen. When I didn’t feel anything I said, aah…am healthy… When I got this pregnancy, I was told, that am sick [meaning HIV+], then I started the treatment (ART). I decided because the health workers convinced me to take it (ART)”* Good adherer, Mityana Hospital

Two delayed ART acceptors were inspired to start ART after receipt of HIV negative test results for their babies.

*“When my child made one and a half months, they tested his blood and he was found okay (meaning HIV negative). Then I started the drugs.”* Delayed ART acceptor, Mityana Hospital

Only one mother, who was a good adherer, reported that her motivation to start ART was to avoid infecting her husband in addition to having an HIV free baby.

### Barriers to initiating ART among HIV infected pregnant and breast feeding women

HIV-related stigma was a major barrier. Some mothers delayed to start ART because of fear of being seen with the ARVs. Others reported throwing away the containers for the drugs and using polythene bags for storage, for fear of being seen with these containers.

*“When I was given the drug, I said how am I going to swallow this, because the people am staying with gossip. I removed the medicines from their container and put them in a polythene bag and then, I threw away the container.”* Good adherer, Mityana Hospital

Some women feared abandonment and domestic violence if their husbands found them in possession of the ARVs. Poor relationships with their spouses were reported to be a barrier to disclosing their HIV positive status and taking ARVs.

*‘I was worried about my husband. If I start swallowing this drug and he sees it, won’t he chase me! Won’t he abuse me!’* Poor adherer, Luwero HCIV

On the other hand, some participants were hesitant to start ARVs because they feared the big size of the tablets. Mothers said the big size of the tablets scared them with some initially failing to start, while others totally refused.

*‘When I took them (ARVs) home, it took me three days to start swallowing, I was looking at the big tablets!…’* Good adherer, Katikamu HCIII

“*I was worried about the size of the tablet…There is no other reason (for not starting treatment), I just feared it*.” Delayed ART acceptor, Masaka Hospital

The length of treatment was also cited as a barrier to starting treatment, particularly with poor adherers and late acceptors. The thought of taking treatment daily for the rest of ones’ life deterred them from starting treatment. Some mothers felt that those who did not adhere to the dosing instructions, would die faster so they did not want to start treatment at all since they were not sure that they would be adherent.

‘*What stopped me from swallowing it; most people say that if you swallow ARVs and you stop, you die!’… I also said, I might not swallow it on time and I also die, so I did not swallow it*.’ Poor adherer, Ssunga HCIII

Some respondents noted that some mothers had refused the ARVs and others had stopped coming to the health facilities due to fear of being tested for HIV. They reported that some mothers come to facilities and receive drugs but throw them away.

‘*Some women when they are given drugs, they throw them away. Because the health worker does not stay at her home, to keep on telling her to swallow the drug. I can go to the hospital and show them an empty tin, but when I have not been taking the drugs. And yet am telling the health workers that am swallowing it. You have to ensure that you go and get more drugs*.’ Poor adherer, Mityana Hospital

*‘There are mothers who come take the drugs and don’t swallow them.’* Delayed acceptor, Kyanamukaka HCIV

### Adherence to ART

Two sub-themes emerged in relation to the theme of ART adherence; facilitators and barriers to good ART adherence.

### Barriers to good adherence to ART

A number of factors were cited for poor adherence including: failure to disclose to spouses, the big size of the tablet and its side effects, organization of health services and stigma. Lack of disclosure was the most common reason for missing ARVs. Most women also reported experiencing disclosure difficulties and consequently struggling to adhere to their treatment.

*‘I time him when he has gone to take a bath, then I swallow it, by the time he comes from bathing, I have already finished.”* Poor adherer, Katikamu HCIII

The disclosure challenges were mentioned by both women who stay with their spouses and those who don’t. One mother who was not staying with her spouse reported not adhering to her treatment for the time when she goes to visit him.

“*I have never forgotten to swallow the medicine(ARVs), but there is one place, where I don’t go with the drug. Only at the taatas’ (the place where her child’s father stays)*…*He said, he cannot get tested…, he cannot take ARVs… Another thing he says that he is not sick*.” When asked why she cannot carry the drugs. She said “*I fear we might get problems… Because I have told him three times to get tested, but for him he says, he cannot get tested’*. Poor adherer, Luwero HCIV

Nearly all mothers (including good adherers) reported substantial challenges with the ARVs. These bad experiences ranged from the big size of the tablet, to its bitter taste and side effects. One mother wondered whether this was a punishment to HIV positive mothers, since other bitter tablets are sugar coated.

“*We women get concerned about our health, the health of our children and our spouses, and if you feel that this medicine is to help you, you try to take it as instructed....,but the main reason which has caused women to stop taking this drug(ARVs), is its strength. As I told you, the tablet is too big. It has challenged a lot of mothers. The tablet is big and the medicine is strong…Eee… we wondered, are we on punishment? Why didn’t those who manufactured this drug make one which is small? Mmh…u see… there are so many drugs for malaria or for what, others are coated and not bitter, but this one, you can throw it in the mouth but before it goes, haa…its bitter and smelling, there is a way it smells! Can’t they put something on it so that it is easy to take?*” Poor adherer, Luwero HCIV

All the mothers reported experiencing side effects ranging from vomiting, headache, drowsiness, body weakness and nightmares. Some of the mothers halted the medication due to side effects. One mother stopped taking ARVs and was only taking Cotrimo xazole, due to the unbearable side effects and the size of the ARVs.

“*The problems I have found in it (ARVs), when you swallow it you dream about the dead! Laughs…when you swallow it you are as if you have drunk Waragi (alcohol)… It gives me drowsiness as if I have drunk waragi……till morning*.” Poor adherer, Ssunga HCIII

“*I had to wake up early to go to school*, *but I failed because I was feeling a headache and nausea…Then I gave up. I decided to take(ARVs) only when am not going to school.”* Poor adherer, Luwero HCIV

Organization of services at health facilities reportedly affected adherence to ART. Some mothers seem to be lost-to- follow-up as they transition from ANC to the chronic care clinic. One mother stopped picking and taking her ARVs and continued only taking Cotrimoxazole which she bought at a private clinic, after discharge from the ANC clinic. Her reason for not returning to pick her ARVs was the fear of being seen at the HIV clinic and thus identified as HIV positive.

“… *Because I have been getting it from maternity, but now they have told me even if the child is one year old, I come here (HIV chronic care clinic)… The problem is that am afraid I might find there people who know me…. I was thinking maybe I start buying it (ARVs)… But they say, even if you don’t swallow the second type (ARVs), that one (Cotrimoxazole) can be able to help you… If I could get it from here (ANC). I can’t go there where many people are… I can’t go there, the clinic is overcrowded.”* Poor adherer, Mityana Hospital

Other factors for poor adherence were forgetfulness, travelling away from home, lack of transport to go to the facility to pick the drugs and lack of food. Some mothers including good ART adherers reported facing challenges in keeping their scheduled appointments for various reasons including lack of transport and other social and family obligations.

“…*I have never missed… I missed like two days. The first time I had gone somewhere, I thought I was not going to sleep there. I had gone on a long journey, for burial, I failed to return and slept there … The second time I was not feeling well, I said I will swallow next day*.’ Good adherer, Katikamu HCIII

Some mothers reported that there are women who refuse to attend clinics due to fear of being known to be HIV+. Some mothers reported missing clinic appointments due to fear of finding people who know them. For example one poor adherer confessed missing clinic appointments due to fear of being identified by friends as HIV positive.

“…*Maybe when I don’t have transport but the other thing, I fear to meet people there. They will talk about me. That is what I fear… I go there (meaning to the health facility) when am worried of meeting there people who know me*.” Delayed acceptor, Katikamu HCIII

Lack of counseling and health education was also raised as a barrier to adherence. Some mothers, lamented that they are not given education and counseling about the treatment. One poor adherer from Mityana attributed her poor adherence to lack of health education about option B+.

‘*Like I have told you, I was not educated… The health worker only tells you your results that you have the virus but go to the other room and find there another health worker. When you reach in the room, the other health worker does not educate you. Tells you, they have told you what has come out of your results and you say yes, the health worker gets you medicine and tells you that you have to take that medicine*.’ Poor adherer, Mityana Hospital

Whereas having an HIV free baby was a motivator to starting ART, to some of the poor ART adherers this was the reason for poorer adherence post-delivery. Some of the reasons for being less adherent after delivery included being too busy taking care of the baby and other responsibilities, and losing the pregnancy or the baby.

*“Musawo(health worker), since we are aware about women’s responsibilities, I won’t lie to you. I have been missing some doses like at times I forget to swallow my dose at night especially when I have too much house work and taking care of the baby’.* Poor adherer, Luwero HCIV

### Facilitators for good adherence

Factors that enhanced good adherence to treatment included: partner/family support, peer support and the outcome of the HIV test results for the baby. Family support for the women, particularly from their spouses for those who had disclosed was reported to facilitate good adherence. This support ranged from being reminded to take their treatment, counselling, to providing them with transport to visit the health facility.

*‘My husband counsels me. When I am away he calls me on phone to remind me when to take the dose.’* Good adherer, Masaka Hospital

Peer support from the family support groups (FSGs) meetings was reported to inspire mothers through continuous counselling. This was reported to enhance adherence and acceptance of ART specifically amongst poor adherers and delayed ART acceptors. The sharing that goes on was cited as an encouragement. Mothers realized that the side effects of the drug are experienced by every mother and they tend to wear off after sometime.

*“…and also in this group (family support group) where we are, it has also continued to help me, because I ask them,…how are the vehicles (night mares)? Some say …mine reduced while others say, my friend mine refused. So I become strong”.* Poor adherer, Luwero Hospital

Receipt of HIV negative results for the baby was also a motivator to good adherence among some mothers. This was said to give them hope and encouragement to take their treatment.

*‘I resumed swallowing the drugs with vigour. I was happy because they had tested the child’s blood and he was healthy (meaning HIV negative).’* Poor adherer, Luwero HCIV

Overall, almost all good ART adherers across all study sites reported self-drive to adhere to their ART medication. Some cited deliberate efforts towards keeping their clinic appointments including reminders or cues to taking drugs, moving with their drugs, and frequently checking on appointment dates to ensure that they do not miss their refills.

*“I always move with my drugs. When it’s time to swallow, I get it and swallow.”* Good adherer, Masaka Hospital

A summary of the women’s experiences regarding barriers and facilitators of uptake and adherence to lifelong ART is presented in Table 2.

**Table 2:** HIV infected pregnant and breastfeeding women’s experiences regarding barriers and facilitators of initiation and adherence to lifelong ART

Theme 1: ART Initiation			
Sub-themes	Good ART adherers	Poor ART adherers	Delayed ART acceptors
*Women’s experience during ART initiation*	-Started ART the same day they tested HIV +, with little effort on counselling and health education -		-Were supposed to start ART the same day they tested HIV+
*Facilitators for initiating ART*	-Desire to have an HIV-free baby		-Receipt of HIV negative results/desire to have an HIV free baby
	-Desire to remain healthy and live longer to be able to take care of their children		-
	-Fear to be recognized as a person living with HIV because of the emaciation		-
	-Health workers motivated	-	-
*Barriers to ART initiation*	-HIV –related stigma		
	-Fear of domestic violence and being abandoned by the spouse		
	-Fear of the big size of the tablets		
	-Fear of the lengthy of the treatment (lifelong)		
	-	-Fear of dying due to the belief that poor adherence to ART kills	
Theme 2: ART Adherence			
Sub-theme			
*Level of adherence*	-Self-drive to adhere to their ART medication -Experiencing moments of non-adherence due to missing doses or clinic appointments -	- Stopped taking ART for some time	-
*Facilitators to ART adherence*	-Family support, especially from the spouse	- Family support	-
	-Peer support by attending family support groups (FSGs)		
	-Receipt of HIV negative results for the baby		
*Barriers to ART adherence*	-Failure to disclose to spouse		
	-Drug related factors.i.e., size and the side effects		
	-HIV-related stigma		
	-Forgetting to take their medication		
	-Travelling away from home		
	-Lack of transport to travel to the health facility to pick drugs		
	-	-Lack of counselling and health education about the treatment	

## Discussion

This study explored pregnant and breastfeeding women’s experiences regarding uptake and adherence to lifelong ART. We identified multifaceted and interconnected facilitators and barriers to ART initiation and adherence. The main incentive for initiating ART for almost all women was the desire to have an HIV-free baby. Fear to disclose their HIV positive status to their male partners, HIV-related stigma and drug related factors were major barriers to initiation of lifelong ART. Adherence to ART was a challenge and the main reasons for poor adherence were; failure to disclose to their male partners, drug-related factors including the big size of the tablets as well as its side effects and stigma. Family support, particularly from the male partners and peer support from the FSGs enhanced adherence. Initiation of lifelong ART was immediate/the same day the mother tested HIV positive and information gaps were prominent.

Whereas having an HIV free baby was a motivator, it bears risks since mothers may go off drugs once they stop breastfeeding or after birth. Indeed some mothers reported not adhering to ART when they lost their babies, after delivery or when they stopped breastfeeding. This is consistent with findings from two studies in Tanzania, where mothers anticipated loss of motivation for taking ART after protecting their babies [12, 13]. In a Tanzanian study, women preferred option B to B+ (lifelong ART), for fear of drug side effects and anticipated loss of motivation after protecting the child [13]. This calls for comprehensive counseling before and after initiating treatment to articulate all the benefits of ART for pregnant women including their own health benefits, HIV transmission to sexual partners and to their babies during subsequent pregnancies [9, 14]. Balancing between the need to urgently initiate treatment and comprehensively preparing women to initiate and sustain treatment will require innovative counseling and support mechanisms to avoid compromising retention and adherence.

Fear to disclose their HIV positive status to male partners, HIV-related stigma and misconceptions about ART were major barriers to initiation of ART. In Uganda, HIV positive mothers are counselled about the importance of disclosure, however it is left to the mother to disclose or not. On the other hand, the women who had disclosed to their partners reported support from the spouse as a facilitator of good adherence. Innovative interventions for assisted disclosure and to ensure male involvement will be critical to ensuring continuity of lifelong ART for women, especially after the ANC phase. This study utilized qualitative approach using IDs. Compared with quantitative surveys, qualitative methods produce more in depth and varied data. A follow-up quantitative study to assess the prevalence of these barriers and facilitators to ART would better inform the design of intervention.

There were various lapses in adherence ranging from not taking the drug at the right time, missing doses and discontinuation for several days. Drug-related factors including the big size and bitterness of the ARV tablets as well side effects were reported by most of the women. Support, particularly from the male partners and FSGs were reported to facilitate adherence. These groups are run by expert clients and mothers and can augment the counselling provided by health workers [15].

One mother reported failing to start on ART after testing HIV positive because she did not feel sick as she had not experienced any symptoms. This is a real concern for lifelong ART especially for women who initiate treatment at high CD4 counts. In Malawi, there was a seven times increase in ART enrollment for pregnant mothers in ANC [7], however women who initiated ART through lifelong ART were 5 times more likely to be lost to follow-up than those who initiated because of low CD4 count [9]. Most women who are started on ART during pregnancy are generally healthy and asymptomatic. Therefore, education to ensure that they appreciate the benefits of early ART for their own health and survival and ongoing counseling for retention and adherence is critical. The gains sought through lifelong ART depend on ensuring good adherence among women [16].

### Study strengths and limitations

Like most qualitative study results, the findings of this study cannot be generalized beyond the context we studied. However, use of in-depth interviews with HIV positive pregnant and breastfeeding mothers facilitated understanding their real life experiences and generates useful lessons for PMTCT programs. In addition, the use of ARV refills as a measure of adherence in classifying women into good and bad adherers has a weakness in that, mothers’ picking ARVs is no guarantee that they were adhering to taking them. It is a rough measure for adherence and is only better in identifying poor adherers than good ones. Therefore, it could have led to some misclassification of the mothers.

## Conclusions

This study has identified numerous facilitators and barriers to both ART initiation and adherence, which can help improve the rollout of lifelong ART among HIV positive pregnant and lactating women. In order to maximize the benefits of lifelong ART it will be necessary to pay attention to adequate preparation for women before initiating treatment and to provide ongoing support for retention and adherence especially after birth and cessation of breastfeeding. Lack of disclosure, drug related factors and minimal male involvement as well as stigma were major barriers to initiation and adherence of lifelong ART.

## Abbreviations

ANC: Antenatal care
FSGs: Family support groups
HC: Health Centre
IDIs: In-depth interviews
